# Coding-complete genome sequences of two strains of canine pneumovirus derived from dogs with upper respiratory disease in the United States

**DOI:** 10.1128/mra.01057-23

**Published:** 2024-01-30

**Authors:** Côme J. Thieulent, Mariano Carossino, Laura Peak, Wendy Wolfson, Ganwu Li, Udeni B. R. Balasuriya

**Affiliations:** 1 Louisiana Animal Disease Diagnostic Laboratory, School of Veterinary Medicine, Louisiana State University, Baton Rouge, Louisiana, USA; 2 Department of Pathobiological Sciences, School of Veterinary Medicine, Louisiana State University, Baton Rouge, Louisiana, USA; 3 Department of Veterinary Clinical Sciences, School of Veterinary Medicine, Louisiana State University, Baton Rouge, Louisiana, USA; 4 Department of Veterinary Diagnostic and Production Animal Medicine, College of Veterinary Medicine, Iowa State University, Ames, Iowa, USA; Katholieke Universiteit Leuven, Belgium

**Keywords:** canine pneumovirus, canine respiratory pathogens, canine infectious respiratory disease complex, *Pneumoviridae*, *Orthopneumovirus*

## Abstract

Canine pneumovirus was detected by RT-qPCR in 2022 from nasal swabs collected from two dogs with upper respiratory disease in a shelter in Louisiana, United States. The genomes from the designated strains CPnV USA/LA/2022/124423 and USA/LA/2022/123696 were sequenced and show the closest similarity to the pneumonia virus of mice J3666.

## ANNOUNCEMENT

Canine pneumovirus (CPnV) is an enveloped virus with a single-stranded negative-sense RNA genome. CPnV is an unclassified virus highly related to the *Orthopneumovirus muris* (genus: *Orthopneumovirus*, family: *Pneumoviridae*, order: *Mononegavirales*) (1, 2). CPnV was first identified in 2010 in the United States from dogs with acute respiratory disease (3) and was subsequently detected in several countries (4–9). Only five complete genome sequences are available in GenBank. Here, we report the coding-complete genome sequences of two CPnV strains, designated as canine_pneumovirus/USA/LA/2022/124423 and canine_pneumovirus/USA/LA/2022/123696.

In August 2022, nasal swabs from two dogs with mild upper respiratory disease, housed together in a Louisiana shelter (USA), were sent for routine diagnostic testing to the Louisiana Animal Disease Diagnostic Laboratory (LADDL). Specimens were collected and nucleic acids were extracted as previously described (10). Both samples were tested positive for CPnV by RT-qPCR (10) and RT-PCR (1). Specimen #124423 was also positive for canine respiratory coronavirus and *Mycoplasma cynos*. Virus isolation was unsuccessful after three blind passages in A-72 cells (ATCC CRL-1542).

The coding-complete sequence of canine_pneumovirus/USA/LA/2022/124423 was obtained through reverse transcription and double-stranded cDNA synthesis, using NEXTflex Rapid RNA-Seq Kit (Bioo Scientific Corp, Austin, TX). Subsequently, the sequencing library was prepared with Nextera XT DNA library preparation kit (Illumina, San Diego, CA) and sequenced on the Illumina MiSeq platform with 250 × 2 read length by following standard Illumina protocols at the Veterinary Diagnostic Laboratory, Iowa State University (Ames, IA). The coding-complete genome sequence of the canine_pneumovirus/USA/LA/2022/123696 could not be generated by direct sequencing due to its high Ct value. Therefore, 10 overlapping cDNA fragments spanning the CPnV genome were synthesized by RT-PCR using QIAGEN OneStep Ahead RT-PCR Kit (QIAGEN). The optimized primer set for CPnV amplification (Table 1) was designed using primalscheme.com (accessed on February 2nd, 2023) using the following reference genomes: KF015281.1, KF015281.1, MK520878.1, and MK520877.1. RT-PCR products were cleaned up using AMPure XP Beads (Beckman Coulter, Brea, CA) and mixed at an equal molar ratio. Library preparation and sequencing were performed as indicated above. Low-quality raw reads and adapters were filtered and trimmed by Seqtk and Trimmomatic-0.36 (11). The filtered reads were tested for quality by FastQC and were assembled utilizing SPAdes 3.13.1-Darwin (12). The resulting contigs were manually curated and refined to obtain the genome sequence according to the reference genome Pneumovirus dog/Bari/100–12/ITA/2012 (KF015281.1). All tools were run with default parameters unless otherwise specified.

**TABLE 1:** Primers used for the amplification of the CPnV genome for sequencing^*b*^

Primer name	Primers sequences (5′−3′)	Size (bp)	Nucleotide position^*a*^	Product size (bp)
CPnV_seq1_F	CCAAACCCACCACTCCKAAA	20	19–39	2,024
CPnV_seq1_R	AAGCACGACRCTGGTRAA	18	2,043–2,026	
CPnV_seq2_F	TTATGGTGGACGAGCTGCATG	21	111–131	1,994
CPnV_seq2_R	AGTTCTCTATTCCTAGGAGCACCC	24	2,104–2,081	
CPnV_seq3_F	CATGCTAGGCCATGCTAGTGTT	22	1,877–1,898	2,007
CPnV_seq3_R	GAGTCAGATAGGGACCCAGTTCT	23	3,879–3,857	
CPnV_seq4_F	CCGATGTGGACCATGCTATAA	21	3,733–3,753	2,023
CPnV_seq4_R	TGGAGCATAGGACACCARTARG	22	5,755–5,734	
CPnV_seq5_F	GCAAYCTGACAGACCAA	17	5,404–5,420	1,941
CPnV_seq5_R	ACAAWGAGCAGAGTTGTTAGTA	22	7,365–3,344	
CPnV_seq6_F	CGCACATTCTTGAAGRCCTCT	21	7,265–7,285	1,999
CPnV_seq6_R	TGTACAACTTRCTGAGCAGATGACC	25	9,263–9,239	
CPnV_seq7_F	TGTCTGGAAGTTTCATACATGTGGT	25	9,030–9,054	2,051
CPnV_seq7_R	ACTGGACATTAGACTTTCCCCCT	23	11,080–11,058	
CPnV_seq8_F	GCTTACARAGGGATAGGTCATAAGC	25	10,844–10,868	2,018
CPnV_seq8_R	TCTGTAACAAAYCCATCATACCATTCT	27	12,861–12,835	
CPnV_seq9_F	GTGCCAAAGCTTCARGAAATAC	22	12,512–12,533	1,991
CPnV_seq9_R	GGGTAAAGCATTTCTATCCTYTCA	24	14,499–14,476	
CPnV_seq10_F	TGGTGTGCGYCTATTGAAGT	20	14,053–14,072	779
CPnV_seq10_R	TYTYAGGGAACTTTYTTTACAACC	24	14,831–14,808	

^*a*^ Nucleotide position based on GenBank Accession number NC_025344.1.

^*b*^ F: Forward; R: Reverse.

**Fig 1:**
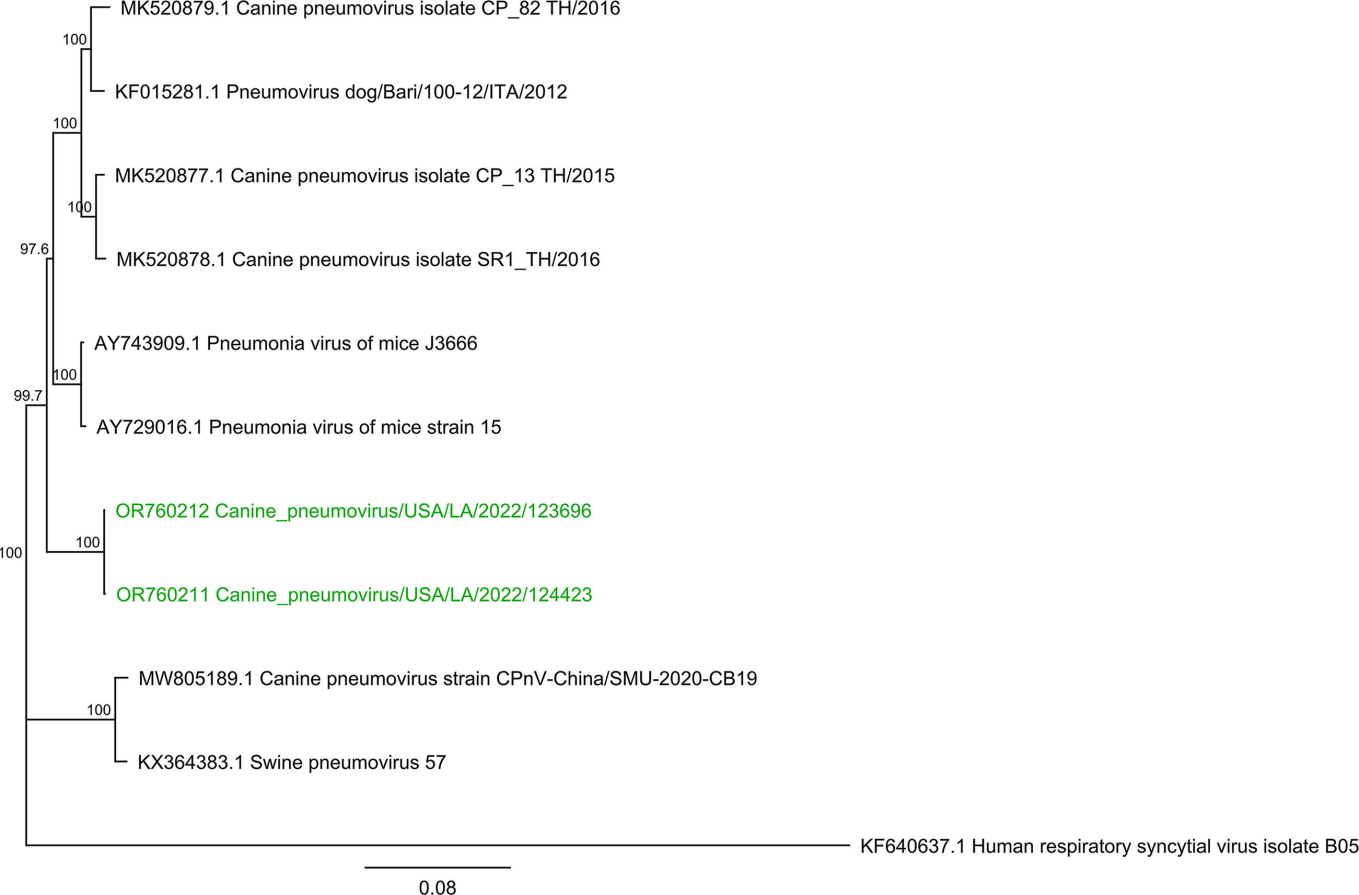
Phylogenetic analysis of the CPnV strains based on the full-length genome with the exclusion of the 5′ untranslated region (UTR) and 3′UTR. The phylogenetic tree included the two CPnV strain sequences obtained in this investigation (OR760211 and OR760212; represented in green), along with five CPnV strains, two *Orthopneumovirus muris* strains, one swine pneumovirus strain, and one *Orthopneumovirus hominis* strain (used as outgroup), obtained from GenBank. Genome alignment and the phylogenetic tree were constructed using Geneious 6.1.8 software. Alignment was performed using the Geneious alignment algorithm, the tree was generated using the neighbor joining method, and the Tamura-Nei model was used for genetic distance estimation. Bootstrapping with 1,000 replicates was performed to assess the robustness of the tree.

The coding-complete genome sequences of canine_pneumovirus/USA/LA/2022/124423 and canine_pneumovirus/USA/LA/2022/123696 were 14,832 bp (GC content: 40.3%) and 14,786 bp (GC content: 40.3%) in length, respectively, with 99.9% nucleotide identity and only nine nucleotide differences. The average coverage depth for each genome was 27.8 and 8889, respectively. While these two CPnV strains form a distinct cluster when compared to the other CPnV and *Orthopneumovirus muris* strains (Fig. 1), they showed the highest nucleotide similarity to pneumonia virus of mice J3666 (NC_006579.1; 94.6% and 94.5%, respectively).

These coding-complete genomes are useful to better understand the genetic diversity and evolution of CPnV, crucial for effective disease management and prevention strategies in canine populations.

## Data Availability

The coding-complete genome sequences of the canine_pneumovirus/USA/LA/2022/124423 and canine_pneumovirus/USA/LA/2022/124423 have been deposited in GenBank under the accession numbers OR760211 and OR760212, respectively. The raw data have been deposited in Sequence Read Archive (SRA) under BioProject ID PRJNA1055968.
